# Supplementary material for: Identifying stochastic oscillations in single-cell live imaging time series using Gaussian processes
Source: PLoS Comput Biol. 2017 May 11;13(5):e1005479. doi: 10.1371/journal.pcbi.1005479 (PMC5444866; doi:10.1371/journal.pcbi.1005479)
Supplement: S1 Table — (PDF) [file pcbi.1005479.s012.pdf]

LLR (-4.5)	Cell number	LLR (-4)	Cell number	LLR (-5)	Cell number
40.9984	cell 13	40.9984	cell 13	40.9984	cell 13
33.8374	cell 10	35.728	cell 15	33.8374	cell 10
31.0297	cell 15	33.8374	cell 10	30.075	cell 15
28.2382	cell 9	28.8639	cell 9	25.4019	cell 9
20.9672	cell 11	22.0586	cell 11	16.6195	cell 11
16.4446	cell 12	18.5871	cell 14	16.4446	cell 12
14.9534	cell 18	16.4446	cell 12	13.4609	cell 18
14.4372	cell 14	14.8987	cell 18	10.8794	cell 17
11.8458	cell 2	12.9729	cell 17	10.6184	cell 44
10.8794	cell 17	12.3385	cell 2	10.2116	cell 2
10.6184	cell 44	10.727	cell 27	10.1728	cell 14
9.14999	cell 27	10.6184	cell 44	7.75873	cell 26
8.75373	cell 8	8.88324	cell 8	7.71507	cell 8
7.87346	cell 26	8.00678	cell 26	5.76766	cell 28
7.21282	cell 19	7.63687	cell 19	5.33541	cell 6
5.64054	cell 6	7.36889	cell 42	4.56567	cell 19
5.56432	cell 28	7.33647	cell 4	4.37002	cell 3
5.5251	cell 4	7.30956	cell 5	4.09345	cell 27
5.45709	cell 42	6.10228	cell 6	4.0546	cell 35
4.89476	cell 5	6.09968	cell 22	3.91524	cell 4
4.63679	cell 3	4.56951	cell 39	3.63046	cell 42
4.24299	cell 22	4.55454	cell 3	3.22251	cell 22
4.08948	cell 35	4.09915	cell 35	2.76633	cell 32
3.25271	cell 39	3.85603	cell 28	2.65737	cell 7
2.77461	cell 7	3.44772	cell 36	2.58263	cell 24
2.72791	cell 32	3.05206	cell 7	2.31273	cell 43
2.68822	cell 36	2.73167	cell 25	2.29182	cell 38
2.60088	cell 24	2.72791	cell 32	2.2635	cell 39
2.39872	cell 25	2.43911	cell 24	2.0984	cell 36
2.36568	cell 43	2.36568	cell 43	1.81549	cell 16
2.22199	cell 38	2.31695	cell 38	1.72817	cell 5
1.87675	cell 16	1.90276	cell 1	1.67971	cell 25
1.19864	cell 30	1.68928	cell 16	0.880202	cell 30
0.811085	cell 37	1.65826	cell 20	0.859294	cell 20
0.80946	cell 20	1.40088	cell 34	0.476036	cell 33
0.708221	cell 1	1.37253	cell 30	0.310482	cell 34
0.624682	cell 33	1.21808	cell 37	0.255126	cell 37
0.494293	cell 31	0.997047	cell 31	0.073898	cell 41
0.310482	cell 34	0.953552	cell 41	0.0212264	cell 29
0.264505	cell 41	0.663787	cell 33	0	cell 1
0.157088	cell 29	0.181401	cell 29	0	cell 21
0	cell 21	1.99944e-05	cell 40	0	cell 23
0	cell 23	0	cell 21	0	cell 31
0	cell 40	0	cell 23	0	cell 40
